# Severe hemolytic anemia and acute renal failure after mitral valve repair associated with non-endothelialization of artificial chordae tendinae: case report

**DOI:** 10.1186/s13019-021-01686-6

**Published:** 2021-10-16

**Authors:** Jing Li, Qun-Jun Duan

**Affiliations:** 1 Department of Cardiology, The Second Affiliated Hospital of Zhejiang University, Hangzhou, China; 2 Department of Cardiovascular Surgery, The Second Affiliated Hospital of Zhejiang University, School of Medicine, #88 Jiefang Road, Hangzhou, 310009 China

**Keywords:** Case report, Hemolytic anemia, Renal failure, Mitral vavle repair

## Abstract

**Background:**

Mechanical hemolytic anemia and acute renal failure are rare complications of mitral valve repair.

**Case presentation:**

We report a unique case of severe hemolytic anemia and severe acute renal failure after mitral valve repair using artificial chordae tendinae. Conservative therapy including plasmapheresis and blood transfusion was not effective. The major cause of the mechanical hemolysis was mild mitral regurgitation originating from the centre of the valve and striking the annuloplasty ring. The hemolytic anemia resolved gradually after the replacement of mitral valve. The new artificial chordae tendinae was found to be completely non-endothelialized in the surgery. Non-endothelialization of artificial chordae tendinae may also play a role in the genesis of mechanical anemia.

**Conclusions:**

The major cause of the mechanical hemolysis was mild mitral regurgitation originating from the centre of the valve and striking the annuloplasty ring. Non-endothelialization of foreign materials might be another mechanism of hemolysis after mitral repair.

## Background

Mechanical hemolytic anemia is a rare complication of mitral valve repair (MVR) [1, 2]. Several mechanisms of such mechanical hemolysis have been reported [1–3]. Although non-endothelialized polytetrafluoroethylene chordae tendinae may contribute to hemolysis, it is extremely uncommon. Acute renal failure (RF) as a serious consequence of mechanical hemolysis after the repair of cardiac valves has been reported only rarely [4–6]. We report an extremely rare case of a man who developed severe hemolytic anemia and severe acute RF shortly after MVR using artificial chordae tendineae. The major cause of the mechanical hemolysis was mild mitral regurgitation. Non-endothelialization of artificial chordae tendinae may also contribute to the genesis of mechanical anemia.

## Case presentation

A 74-year-old man was admitted to our hospital for severe mitral regurgitation. Further examination revealed prolapse of the anterior leaflet and rupture of the chordae tendineae. Baseline serum creatinine level was 102.5 μmol/L, and hemoglobin level was 12.6 g/dL. He underwent surgery for chordal replacement with expanded polytetrafluoroethylene chordae tendineae. And mitral annuloplasty was performed with a flexible annuloplasty Medtronic Duran ring. Intraoperative transesophageal echocardiography and postoperative transthoracic echocardiography demonstrated no residual mitral regurgitation. The patient was discharged in good condition one week after surgery. At the time of discharge, serum creatinine level was 87.5 μmol/L.

**Fig. 1:**
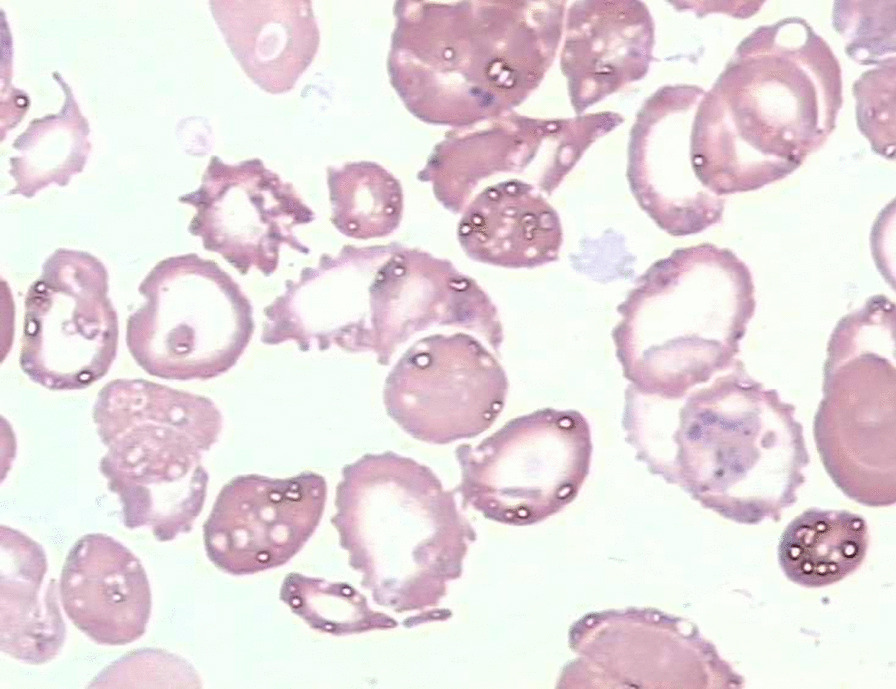
Peripheral blood smear demonstrated schistocytosis and red cell fragmentation

**Fig. 2:**
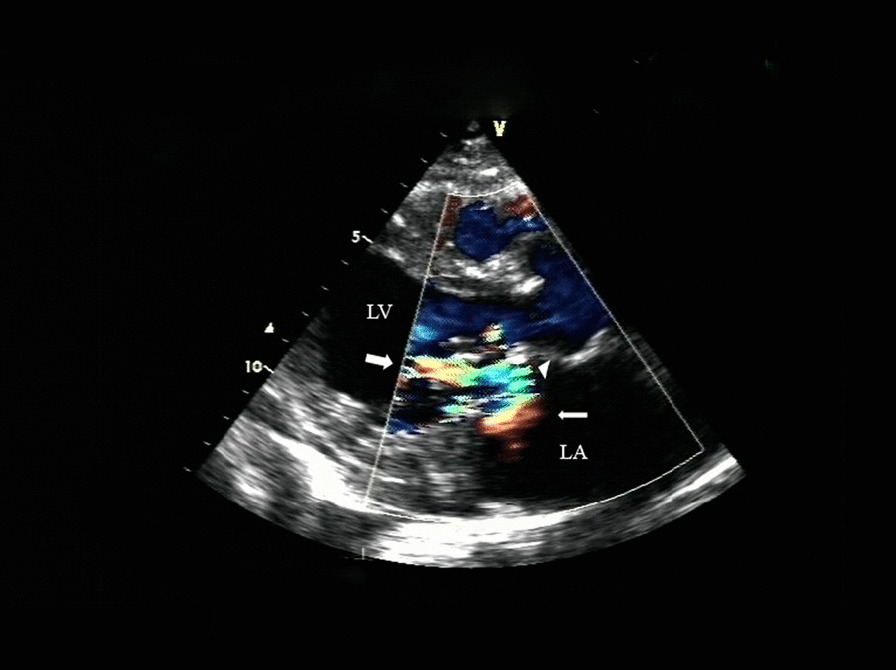
Transthoracic echocardiography showing a jet of mitral regurgitation (large arrow) which immediately collides with the annuloplasty ring (arrowhead), redirecting the jet at a right angle into the central LA (small arrows). LA, left atrium; LV, left ventricle

**Fig. 3:**
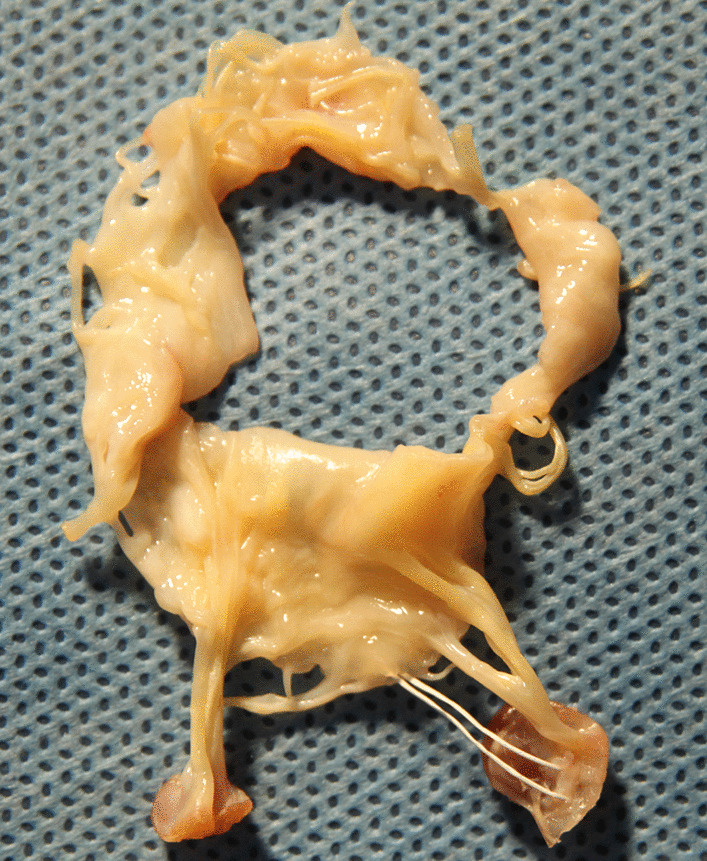
The artificial chordae tendinae is completely non-endothelialized

Seven weeks after surgery he presented with anemia and “dark urine” and was admitted to our hospital. Investigations showed haemoglobin 4.5 g/dL, hematocrit 12%, undetectable haptoglobin, reticulocyte count 9%, slightly depressed platelet count 75,000/μL, serum lactic dehydrogenase 3600 IU/L, and total bilirubin of 69.86 μmol/L (indirect of 57.1 μmol/L). Peripheral blood smear demonstrated mechanical hemolysis with schistocytosis and red cell fragmentation (Fig. 1). Creatinine levels ranged from256 to 362 μmol/L. Coomb’s test, Ham test and sugar water test were negative. Expression of CD55 and CD59 for paroxysmal nocturnal hemoglobinuria was normal. Drug-induced hemolytic anemia was not apparent, because there was no reduction of hemolysis after stopping the administration of any drug. On transthoracic echocardiography, mitral valve jet was found and judged to be mild, originating from the centre of the valve and striking the annuloplasty ring (Fig. 2). The velocity of the mitral regurgitation jet was 2.3 m/s. The investigation did not show any evidence of a structural defect of the valve or the annuloplasty ring. Predonisolone for the possible microangiopathy was used. Three times of plasmapheresis and blood transfusion of 15 units red blood cells were required. After these treatments, the anemia and dark urine did not improve, with a creatinine level at 256 μmol/L. Based on previous experience with hemolytic patients following MVR and useless of aggressive conservative therapy, we decided to do reoperation for intracardiac inspection and mitral valve replacement after a detailed communication with the patient, despite RF. During the intraoperative inspection, there was no ring dehiscence or suture-related tear of the valve tissue. The annuloplasty ring was well endothelialized. Chordal attachments were intact. However, the new artificial chordae tendinae was completely non-endothelialized (Fig. 3). The plasty ring was excised and the mitral valve was replaced with a 27-mm bileaflet prosthesis (Edwards Lifesciences). The hemolytic anemia resolved gradually after the reoperation. But the RF persisted after surgery and the patient was transferred to the department of hematology for hemodialysis. Three months later, the LDH level gradually fell to 224 U/L. His hemoglobin level was stable at 95 g/dL with 3% reticulocytes. Serum creatinine level was 80.4 μmol/L.

## Discussion

Hemolysis occurs in the majority of patients with mechanical prosthetic valvular replacement. On the contrary, hemolysis following MVR and annular ring placement is uncommon because there are no moving mechanical parts or prosthetic leaflets [6]. Mechanisms of hemolysis following MVR that have been suggested include the ‘whiplash motion’ of the residual free-floating chordae tendineae, collision of the regurgitant jet into the prosthetic ring, central jets colliding with the atrial wall, fragmentation of the regurgitant jet by a dehisced annuloplasty ring, nonendothelialization of annuloplasty ring, and rapid acceleration of a jet through a small para-ring channel [2, 3, 7–9]. This case is a unique case. The major cause of the mechanical hemolysis was mild mitral regurgitation which originated from the centre of the valve and striking the annuloplasty ring. And we suspect that non-endothelialization of artificial chordae tendinae may be another cause.

Although intravascular hemolysis is common after placement of a prosthetic valve or valve repair, there have been only few reported cases of severe acute RF in the literature [4, 6]. This case was an uncommon case. It developed shortly to severe acute RF. Plasmapheresis and blood transfusion were not effective. Pharmacological approaches with the use of antioxidants or agents directed at increasing red blood cell flexibility or decreasing hydrodynamic shear forces were also invalid. Hemodialysis was required finally. Hence, acute RF from hemolysis after MVR is a rare, but serious, complication that should be recognized because chronic kidney disease may ensue [6].

Expanded polytetrafluoroethylene is a linear, nonabsorbent monofilament polymer that has been used in cardiovascular surgery patches and sutures for many years and has many documented advantages over other suture materials [4, 10]. MVR surgery with implantation of expanded polytetrafluoroethylene chordae tendinae has been offered as a safe and effective surgical alternative for mitral valve. However, it has been reported that expanded polytetrafluoroethylene suture was calcified and ruptured and that no connective tissue or calcium deposits was found in the suture material [4, 10, 11]. We report a unique case of completely non-endothelialized expanded polytetrafluoroethylene. We suspect that non-endothelialization of the expanded polytetrafluoroethylene might play a role in the genesis of mechanical hemolysis. But it is difficult to identify whether it is primary or secondary.

Residual mitral regurgitation was the major cause to mechanical hemolysis. High velocity mitral regurgitant jet could denude the endothelium, expose the expanded polytetrafluoroethylene surface, prevent complete endothelialization of expanded polytetrafluoroethylene and increase the risk of hemolysis [2]. In the case presented here, the velocity of the regurgitant jet as assessed by Doppler analysis was 2.3 m/s. Such a high-speed jet could cause turbulence and increased trauma. Although many reported cases had high grade mitral regurgitation, echocardiography only showed mild mitral regurgitation and no other structural findings in this case [1]. Therefore, though rarely, the clinician should have an index of suspicion for mechanical hemolysis even if the mitral regurgitation is mild or trivial.

## Conclusion

We present an uncommon case of severe hemolytic anemia accompanied by severe acute RF after MVR using artificial chordae tendinae. The major cause of the mechanical hemolysis was mild mitral regurgitation originating from the centre of the valve and striking the annuloplasty ring. Non-endothelialization of foreign materials might be another mechanism of hemolysis after mitral repair.

## Data Availability

The datasets used and/or analysed during the current study are available from the corresponding author on reasonable request.

## Abbreviations

MVR: Mitral valve repair
RF: Renal failure
